# PI3K/Akt in IPF: untangling fibrosis and charting therapies

**DOI:** 10.3389/fimmu.2025.1549277

**Published:** 2025-03-31

**Authors:** Janki Bhatt, Alessandra Ghigo, Emilio Hirsch

**Affiliations:** ^1^ Department of Molecular Biotechnology and Health Sciences, Molecular Biotechnology Center “Guido Tarone”, University of Turin, Turin, Italy; ^2^ Kither Biotech S.r.l., Turin, Italy

**Keywords:** idiopathic pulmonary fibrosis, PI3K/AKT, inflammation, fibrosis, senescence, PI3K inhibitor, PI3K inhibition

## Abstract

Idiopathic Pulmonary Fibrosis (IPF) is a chronic, progressive lung disease characterized by abnormal epithelial repair, persistent inflammation, and excessive extracellular matrix deposition, leading to irreversible scarring and respiratory failure. Central to its pathogenesis is the dysregulation of the PI3K/Akt signaling pathway, which drives fibroblast activation, epithelial-mesenchymal transition, apoptosis resistance, and cellular senescence. Senescent cells contribute to fibrosis through the secretion of pro-inflammatory and profibrotic factors in the senescence-associated secretory phenotype (SASP). Current antifibrotic therapies, Nintedanib and Pirfenidone, only slow disease progression and are limited by side effects, highlighting the need for novel treatments. This review focuses on the role of PI3K/Akt signaling in IPF pathogenesis, its intersection with inflammation and fibrosis, and emerging therapeutic approaches targeting molecules along this pathway.

## 1 Introduction

Idiopathic Pulmonary Fibrosis (IPF) is a chronic, progressive fibrosing interstitial pneumonia of unknown origin, characterized by a progressively worsening dyspnea and declining lung function (1, 2). It is a fatal age-related disorder, predominantly occurring in males, with a median survival age of 2-5 years after diagnosis (3, 4). Although considered rare, existing data indicates that the global occurrence of IPF rivals several cancer types, including stomach, liver, testicular, and cervical cancers (5), with approximately 40,000 new cases diagnosed annually in Europe alone (6), and its incidence doubling with each successive decade after the age of 50. The fatality of the disease, combined with its increasing prevalence and the subsequent strain on healthcare resources, highlights the urgency of addressing IPF treatment.

**Figure 1:**
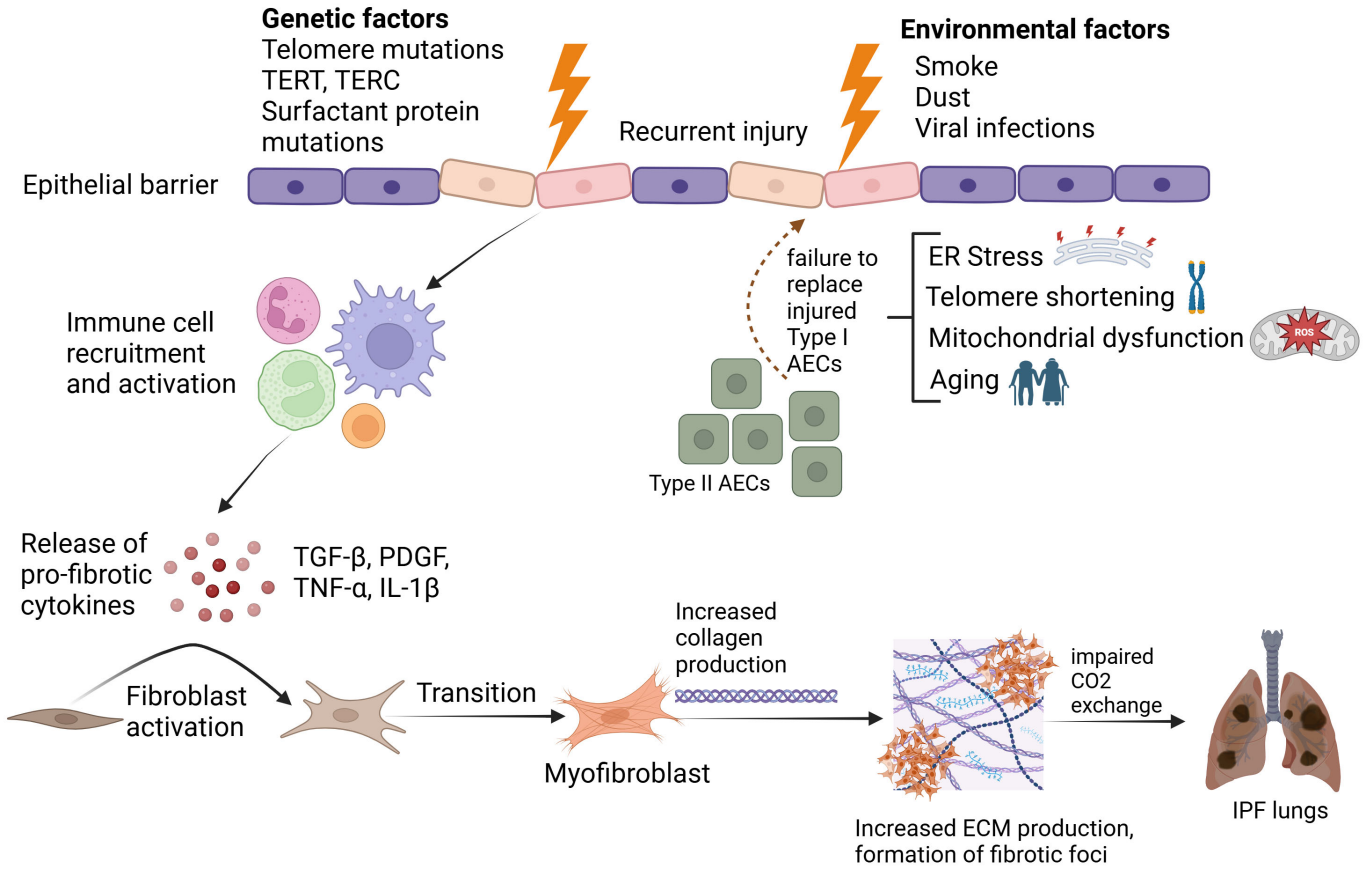
Pathophysiological mechanisms underlying Idiopathic Pulmonary Fibrosis. Created in BioRender. Ghigo, A. (2025) https://BioRender.com/2jn4ve5.

Historically, IPF was understood to be an inflammation driven disease that eventually progressed to fibrosis. However, this concept was challenged by two pivotal clinical trials. The first, INSPIRE, found that patients treated with Interferon-γ did not experience significant clinical benefits. The second, PANTHER, evaluated a combination of three immunosuppressive agents (prednisone, azathioprine, and N-acetylcysteine) and revealed increased risks of death and hospitalization compared to placebo. (1, 7). These trials marked a paradigm shift in our understanding of IPF, suggesting that fibrosis, rather than inflammation, is central to IPF pathophysiology. Fibrosis in IPF is characterized by fibroblast activation, excessive collagen deposition, leading to the distortion of lung architecture and impaired gas exchange (**Figure 1**). This fibrotic process is driven by the aberrant activation of fibroblasts and myofibroblasts, which secrete collagen and other extracellular matrix proteins, resulting in the thickening and stiffening of the lung tissue. In the context of these findings, two antifibrotic therapies, Nintedanib and Pirfenidone, have been developed and approved for the treatment of IPF (8, 9). However, these drugs only slow down disease progression (10) and up to 40% patients discontinue treatment citing gastrointestinal, dermatological or liver-associated adverse drug reactions (11). Hence, lung transplantation remains the last resort for IPF patients, which is impractical for a majority of cases, considering factors such as age and comorbidities (4).

At a molecular level, the transition from inflammation to fibrosis as the focus of IPF research was further substantiated by studies that identified key signaling pathways involved in fibrogenesis (12). Among these, the PI3K/Akt pathway is involved in vital cellular processes such as survival, growth, proliferation, metabolism, apoptosis and angiogenesis. PI3Ks are lipid kinases divided into three classes – Class I, Class II and Class III. In mammalian cells, Class I PI3K catalytic subunits catalyze the phosphorylation of PtdIns-4,5-P_2_ (PIP2) to generate PtdIns-3,4,5-P_3_ (PIP3). Upon phosphorylation, PIP3 recruits two pleckstrin homology domain-containing kinases: the serine threonine kinase, Akt, and phosphoinositide-dependent kinase 1 (PDK-1). Akt undergoes conformational changes on direct binding to PIP3, exposing two of its amino acid sites, serine 473 and threonine 308, for phosphorylation by mammalian target of rapamycin complex 2 (mTORC2) and PDK1, respectively (13, 14). Once fully activated, Akt readily phosphorylates its downstream effectors such as mammalian target of rapamycin (mTOR), nuclear factor-κB (NF-κB), and p70 ribosomal protein S6 kinase (p70S6K), contributing to various cellular processes. PI3K/Akt signaling mediates key cellular functions such as proliferation and survival, bridging the mechanistic overlap between tumorigenesis and fibrotic progression of IPF. Understanding its regulation and potential for therapeutic targeting is critical to advancing IPF research and treatment strategies. Therefore, this review focuses on the role of the PI3K/Akt pathway in IPF pathogenesis, examining its multifaceted interactions and elucidating how dysregulation within this signaling axis contributes to the progression of fibrotic lung disease. Additionally, the review explores existing and emerging therapeutic avenues targeting key molecules along the PI3K pathway, discussing novel exploratory strategies for IPF treatment.

## 2 The PI3K/Akt signaling pathway: senescence, fibrosis and the PI3K signaling nexus

The PI3K/Akt signaling axis integrates key processes of cellular senescence, linking hallmark features of aging—such as telomere attrition, mitochondrial dysfunction, and impaired autophagy—to the molecular pathways underlying IPF pathogenesis. The association between IPF and aging is evidenced by the characteristic features of aging that are also observed in IPF, including impaired autophagy, DNA damage, mitochondrial dysfunction and telomere attrition. Telomere shortening triggers senescence, and is also an important risk factor for IPF, particularly familial IPF (15). Mutations in hTERT or hTR result in shorter telomeres and this telomere attrition in murine alveolar epithelial type 2 cells (AEC), but not mesenchymal cells, has been shown to promote fibrosis (16, 17). Lung fibroblasts from IPF patients also exhibit a senescent phenotype characterized by shorter telomeres and mitochondrial dysfunction (18). However, cellular senescence typically serves as a protective mechanism by limiting excessive cell proliferation, hence its role in promoting fibrosis in IPF appears paradoxical. Studies have shed light on the mechanisms through which senescence contributes to fibrosis. (19, 20). Telomere shortening exacerbates AEC dysfunction, while mitochondrial impairment and increased reactive oxygen species (ROS) drive the activation of pathways such as PI3K/mTOR. In the context of aging and IPF, reduced or lost PTEN expression induces Akt-dependent alveolar epithelial senescence (21). Akt hyperactivation drives apoptosis resistance and mTOR activity leads to inhibition of autophagy, factors which are necessary for fibrogenesis (22). This process leads to the upregulation of senescence markers including senescence-associated β-galactosidase, p21, p16, and p53, along with the emergence of a senescence-associated secretory phenotype (SASP). SASP is characterized by the secretion of pro-inflammatory and pro-fibrotic cytokines, including interleukin-6 (IL-6), interleukin-8 (IL-8), and TGF-β.

The emergence of SASP, driven by upregulated senescence markers, has been closely linked to PTEN loss and Akt hyperactivation to senescence, as demonstrated in bleomycin-induced models where Akt2 knockdown mitigated the senescence phenotype (21). In IPF, AEC are particularly prone to this senescent phenotype due to persistent PI3K/Akt activation in the absence of adequate PTEN function (21, 23, 24). Although SASP production occurs in both fibroblasts and AECs, it is the SASP from AECs that appears to be critical for the progression of fibrosis. This dysfunctional feedback loop of senescence, SASP production, PI3K signaling mediated fibroproliferation and apoptosis resistance, ultimately accelerates fibrosis, making the PI3K/Akt axis a critical target for therapeutic intervention in IPF by addressing the underlying mechanisms of senescence and fibrosis (15, 24, 25).

### 2.1 The effectors upstream of PI3K/Akt and their roles in IPF

Upstream and downstream effectors of the PI3K/Akt signaling pathway drive IPF progression, mediating the interplay between pro-fibrotic stimuli and cellular responses. Upstream, growth factors and cytokines such as TGF-β, PDGF, and VEGF aberrantly activate the pathway, driving processes like fibroblast proliferation and extracellular matrix production. Downstream, PI3K/Akt signaling regulates key effector mechanisms, including myofibroblast differentiation, epithelial-mesenchymal transition (EMT), and metabolic reprogramming, which together perpetuate the fibrotic environment. Understanding these interactions reveals how dysregulated PI3K signaling orchestrates IPF pathogenesis.

In IPF, damaged alveolar epithelial cells, alveolar macrophages and fibroblasts release profibrotic cytokines such as TGF-β, Platelet-Derived growth factor (PDGF), Connective Tissue Growth Factor (CTGF) Vascular Endothelial Growth Factor (VEGF), and fibroblast growth factor (FGF) which aberrantly stimulate the PI3K/Akt pathway (26–28), hence perpetuating the cycle of injury and dysregulated repair. PDGF, a key target of Nintedanib, plays a critical role in stimulating fibroblast proliferation and collagen deposition via the PI3K/Akt pathway (29). TGF-β is the primary orchestrator of lung fibrosis, driving fibroblast activation, differentiation, and Extra Cellular Matrix (ECM) deposition. TGF-β also activates CTGF expression by primary AECs (30), which further stimulates EMT and upregulates ECM deposition. CTGF in turn induces activation of TGF-β as well as VEGF in a positive feedback loop (30, 31). This loop can be broken by CTGF inhibition, but CTGF expression by type 2 AECs and active fibroblasts is more prominent in the early stages of fibrosis and reduced in the later stages (32). This could explain why a phase 3 study of a CTGF inhibitor, Pamrevlumab, showed no clinical benefits compared to placebo (33). Apart from its direct effect on CTGF, TGF-β, increases hypoxia-inducible factor-1α (HIF-1α) expression via Smad signaling, thereby upregulating VEGF gene expression (34, 35). Akt activation also induces the upregulation of HIF-1α expression, which subsequently increases VEGF levels, promoting angiogenesis (36). Growth factors play a complex and sometimes isoform-specific role in fibrosis, with certain variants promoting fibrotic responses while others exhibit antifibrotic properties. For instance, VEGF165b has been shown to inhibit fibrosis, in contrast to VEGF165a, which promotes fibrotic processes (37, 38), implying that a more isoform-specific approach is required to yield substantial clinical benefits. Similarly, FGF was traditionally considered profibrotic, but newer findings suggest a preventative and therapeutic role. FGF1 attenuates TGF-β1 induced fibrosis by decreasing e-cadherin expression and inhibiting myofibroblast differentiation (39), FGF2 suppresses collagen production and fibroblast differentiation (40) while FGF7 and 10 improve lung repair and prolong survival in mouse models (41).

Growth factor activation of PI3K promotes cell survival, but PTEN counterbalances it by dephosphorylating PIP3 to inhibit Akt (42). Myofibroblasts extracted from IPF biopsies show reduced PTEN expression alongside Akt upregulation, and in bleomycin-induced mouse models, PTEN deficiency has been shown to exacerbate fibrosis, resulting in a 55% increase in collagen content compared to wild-type mice (43, 44). PTEN suppression and aberrant activation of Akt can result in decreased autophagic activity, creating an environment that promotes fibroblast proliferation and apoptosis resistance. When IPF fibroblasts interact with type I collagen—a major component of the ECM in fibrotic tissues— Akt activity remains high while autophagy markers, such as LC3-II, are suppressed (45–47).

### 2.2 The effectors downstream of PI3K/Akt and their roles in IPF

A recent groundbreaking study in fibroblast lineage tracing identified a fibrotic fibroblast subset and two distinct inflammatory fibroblast subsets that drive both fibrosis and chronic inflammation. The fibrotic fibroblasts, marked by high Collagen Triple Helix Repeat Containing 1 (CTHRC1) expression, are central to active fibrotic foci and serve as the predominant producers of collagen and extracellular matrix (ECM) components, thereby driving fibrosis. The inflammatory fibrotic subset 1 localizes near the fibrotic foci, while inflammatory fibrotic subset 2 resides within the fibrotic foci, toward the interstitial side (48, 49). The PI3K/Akt signaling pathway has been shown to promote persistent fibrosis by driving apoptosis resistance and fibroblast proliferation, suggesting that this pathway may contribute to the maintenance and expansion of the fibrotic fibroblast population. Downstream of PI3K/Akt, Akt phosphorylates and inactivates FOXO3a, impairing its ability to regulate apoptosis, thereby allowing fibroblast accumulation and transition into myofibroblasts, further exacerbating fibrosis (50–52). While CTHRC1-expressing fibrotic fibroblasts are central to fibrosis progression, Tsukui et al. demonstrated that eliminating this population alone was insufficient to attenuate fibrosis, highlighting the involvement of additional fibroblast subsets. Beyond fibroblast proliferation, fibrosis progression also involves epithelial remodeling through epithelial-mesenchymal transition (EMT), a key process in which epithelial cells lose their identity and acquire mesenchymal traits, further contributing to the expansion of pro-fibrotic fibroblast populations. EMT is characterized by the loss of epithelial markers like E-cadherin and upregulation of mesenchymal markers such as α-smooth muscle actin (α-SMA), vimentin, and fibronectin (53). Akt promotes EMT by activating transcription factors like Snail, ZEB1/2, Twist, and LEF-1, which repress E-cadherin and induce mesenchymal marker expression (54). Akt also phosphorylates GSK3-β, preventing β-catenin degradation. This stabilizes and translocates β-catenin to the nucleus, driving EMT-associated gene transcription (55, 56). *In vivo*, Akt1−/− mice show resistance to hypoxia-induced pulmonary fibrosis, directly linking Akt inhibition to reduced fibroblast activity and apoptosis resistance (57). Considering that EMT-derived fibroblasts may also contribute to the fibrotic fibroblast pool, the PI3K/Akt axis appears to play a central role in sustaining fibrotic fibroblast populations through both direct fibroblast proliferation and EMT-driven mesenchymal transition.

## 3 PI3K/Akt signaling based strategies to treat IPF

Over the past two decades, extensive clinical trials have aimed to identify effective treatments for IPF. However, most investigational therapies have failed to demonstrate significant efficacy, highlighting the complex and challenging nature of this disease (58, 59). This challenge arises in part from the poorly understood mechanisms driving IPF progression and the difficulty of reversing established fibrosis. Current approved therapies, Pirfenidone and Nintedanib, offer some benefit in slowing disease progression but are far from optimal in halting or reversing the fibrotic processes.

### 3.1 Commonalities between lung cancer and IPF

IPF and non-small cell lung cancer (NSCLC) share molecular, cellular, and genetic similarities, including mutations in genes like p53, KRAS, and p21. Shared genetic predispositions, such as mutations in SFTPA1 and SFTPA2, lead to endoplasmic reticulum stress, impaired protein secretion, and apoptosis, driving fibrosis in IPF and tumorigenesis in lung cancer. Both diseases exhibit epigenetic changes, such as deregulated noncoding RNAs like miR-21, and involve common processes like EMT and apoptosis resistance. Abnormal activation of pathways like Wnt/β-catenin and PI3K/Akt further contributes to metaplasia, hyperproliferation, fibrosis, and tumor growth (60–62).The PI3K/Akt pathway is central to both diseases, promoting cell survival, proliferation, and resistance to apoptosis in NSCLC, and driving fibroblast proliferation, ECM deposition, and apoptosis resistance in IPF. Its influence on EMT, a shared process in fibrosis and cancer, facilitates epithelial marker loss and mesenchymal trait acquisition, fueling fibrosis and tumor invasion.

Despite these overlaps, IPF differs from cancer as it lacks monoclonality and metastatic potential. Cancer often begins in one organ and spreads, while IPF is a bilateral disease from early stages (63, 64). These differences necessitate tailored treatment strategies for IPF. Drugs like Nintedanib, originally developed for cancer, target pathways common to fibrosis and tumorigenesis, slowing disease progression in both NSCLC and IPF. Similarly, Pirfenidone inhibits EMT and tumor stroma, addressing fibrosis and cancer metastasis. However, both treatments have dose-limiting toxicities, highlighting the urgent need for new therapies in IPF.

### 3.2 PI3K/Akt/mTOR inhibitors to target IPF

Based on the overlapping metabolic profiles of IPF and lung cancer, Omipalisib was the first PI3K/mTOR inhibitor originally developed for solid tumors, and later repurposed for IPF. Pharmacokinetic studies with Omipalisib showed a decrease in glucose uptake and an acceptable tolerability profile; however, patients also experienced adverse events like gastrointestinal disturbances and hyperglycemia, which are consistent with the expected toxicities of PI3K/mTOR inhibition (65, 66). These findings highlight the therapeutic promise of PI3K/mTOR inhibitors for IPF while emphasizing the need for safer alternatives.

#### 3.2.1 Repurposed PI3K/mTOR inhibitors

Prior to Omipalisib, Everolimus, a selective mTORc1 inhibitor, was tested in 89 IPF patients but worsened disease progression, possibly due to its partial inhibition of 4E-BP1, a key driver of TGFβ1-induced collagen production (67, 68). Newer dual mTORc1/mTORc2 inhibitors, such as Sapanisertib and Vistusertib, have shown promise in reducing TGF-β1-induced EMT, fibrosis markers, and proinflammatory cytokines like tumor necrosis factor-alpha (TNF-α), interleukin-1 beta (IL-1β) and interleukin-6 (IL-6)in preclinical models (69–71). Their clinical success, however, depends on balancing efficacy and safety, a challenge for PI3K/mTOR inhibitors. Current limitations of mTOR inhibitors suggest a need for better selectivity and targeted delivery. A key question that remains unaddressed in IPF is whether isoform selective PI3K inhibition, such as with drugs like Alpelisib that selectively target PI3Kα, could provide improved clinical benefits. If isoform-specific inhibition is sufficient to halt fibrosis progression, a PI3Kα degrader like WJ112-14, which selectively tags the alpha isoform for degradation, could offer greater efficacy over reversible kinase inhibition by ensuring sustained depletion of PI3Kα levels, although this remains to be clinically validated (72). Notably, a dual PI3K-δ/γ inhibitor, Duvelisib, indicated for Chronic Lymphocytic Leukemia, was recently shown to attenuate fibrosis *in vivo* and *in vitro* (73). Although these findings are clinically relevant, delivery, dosage and toxicity remain key considerations given the heterogenous nature of this disease. In contrast to isoform-specific inhibition, a first-in-class pan-PI3K and dual mTORc1/mTORc2 inhibitor, Gedatolisib, was shown to have superior potency and efficacy over alpelisib, capivasertib (AKT inhibitor), and everolimus in breast cancer cell lines. Currently, a global combinatorial phase 3 study with Gedatolisib is underway for patients with HR+/HER2− ABC mutations (74). The results of this trial may inform future studies exploring the potential of pan-PI3K/dual mTOR inhibitors in the treatment of IPF.

#### 3.2.2 Novel PI3K/mTOR inhibitors

While drug repurposing remains a promising strategy, significant advancements have also been made in developing novel PI3K/mTOR inhibitors designed specifically for IPF pathophysiology. These drugs provide unique mechanisms of action and modes of administration, which could potentially leading to greater clinical benefits over existing drugs. Campa et al. developed KITCL27, as an inhalation-based prodrug pan-PI3K inhibitor designed to be activated only after hydrolysis inside target cells. Preclinical studies of KITCL27 in bleomycin-induced mouse models showed that inhaled CL27c effectively reduced lung fibrosis by decreasing collagen deposition and downregulated key pro-fibrotic markers, including TGF-β1, CTGF, Collagen type I (Col1a1), and matrix metalloproteinase-2 (MMP2) (75). Another innovative approach to PI3K inhibition was the development of FAPL-PI3Ki1 by Hettiarachchi et. al., which is a PI3K inhibitor conjugated to a ligand that targets fibroblast activation protein (FAP), a protein exclusively expressed by collagen-producing myofibroblasts. This FAPL-PI3Ki1 demonstrated reduced collagen deposition, decreased the expression of fibrotic markers such as α-SMA and Col1A1, and improved survival in bleomycin-induced mice (76).

#### 3.2.3 Akt inhibitors

Considering the ineffectiveness of existing drugs, the toxicity of PI3K inhibitors and the role of Akt hyperactivation in fibrosis progression, targeting Akt through pharmacological inhibition offers a new therapeutic approach for IPF. Given the extensive research and development that has already been conducted on Akt inhibitors for cancers, their pharmacokinetics, safety profiles, and molecular interactions are well understood, making them strong candidates for repurposing in IPF (**Table 1**) (57, 77). Notably, Akt inhibitors like Triciribine and MK-2206 have already been tested in bleomycin-induced mouse models of IPF (78, 79) supporting their use and development for IPF.

**Table 1:** Summary of Akt inhibitors used as monotherapies for cancers, with the potential for repurposing in IPF.

Drug Name	Original Indication	Mechanism of Action	Relevance in IPF
**Triciribine**	Solid Tumors and Hematological Malignancies	ATP-competitive Akt inhibitor	In bleomycin-induced mouse models, Triciribine reduced pro-inflammatory cytokines (TNF-α, IL-6) and fibrosis markers (TGF-β, collagen I).
**MK-2206**	Solid Tumors including non-small cell lung cancer (NSCLC)	Allosteric Akt inhibitor	In bleomycin-induced mouse models, MK-2206 reduced inflammatory cytokine release (TNF-α, IL-6) and decreased ECM production in fibroblasts.
**Perifosine**	Multiple Myeloma and Other Cancers	Allosteric Akt inhibitor	In radiation-induced lung injury models, Perifosine inhibited the PI3K/Akt pathway in Sox9-expressing lung epithelial cells.
**Afuresertib**	Solid Tumors	ATP-competitive Akt inhibitor	Clinical evidence required to study its use in IPF
**Ipatasertib**	Prostate Cancer, Breast Cancer	ATP-competitive Akt inhibitor	Further research needed to explore antifibrotic effects in IPF relevant models
**Capivasertib**	Prostate Cancer, Breast Cancer	ATP-competitive inhibitor	Further research needed to explore antifibrotic effects in IPF relevant models
**Uprosertib**	Solid Tumors including NSCLC	ATP-competitive Akt inhibitor	Further research needed to explore antifibrotic effects in IPF relevant models, could be repurposed considering its effects in NSCLC

Similar to oral PI3K inhibitors, the development of Akt inhibitors faces challenges due to adverse effects such as diarrhea, nausea, and skin rashes, as observed in clinical trials for solid tumors. ATP-competitive inhibitors like ipatasertib and capivasertib struggle with selectivity issues within the AGC kinase family, leading to off-target effects. In contrast, allosteric inhibitors that bind to the Pleckstrin Homology domain of Akt offer greater specificity for Akt. However, these allosteric inhibitors have shown limited clinical efficacy and require optimization before they can be considered viable options for IPF treatment.

#### 3.2.4 Akt degraders

A novel alternative to pharmacological inhibition of Akt is to reduce intracellular Akt levels via protein degradation. This strategy has led to the development of Akt degraders, which are proteolysis targeting chimeras linked to Akt inhibitors. INY-03-041 is a pan-Akt degrader that combines GDC-0068, a pan-Akt inhibitor, with Lenalidomide, an immunomodulatory drug that recruits Cereblon (CRBN) and brings it into close proximity to Akt. CRBN acts as a substrate adaptor, guiding Akt to the E3 ubiquitin ligase complex, where Akt is tagged with ubiquitin and subsequently degraded by the proteasome (80, 81). To date, there are only a handful of Akt degraders currently under development for solid tumors, including INY-03-041, MS21, MS143 and MS5033. Although they are yet to be repurposed for clinical studies in IPF, Akt degraders exhibit a longer half-life and a more prolonged pharmacological effect compared to their constituent Akt inhibitors alone (81), which could provide added benefits in terms of dose reduction. However, the on-target toxicities already observed with pharmacological AKT inhibitors, combined with the increased potency of AKT degraders suggest that AKT degraders may have a smaller therapeutic window. Therefore, it will be critical to explore dosages to achieve a balance between safety and efficacy for both, cancers and IPF.

### 3.3 Targeting other mediators along the PI3K/Akt pathway

Since the approval of Nintedanib, several tyrosine kinase inhibitors have been repurposed for IPF with mixed outcomes. Imatinib, a PDGFR and TGF-β inhibitor, was discontinued due to lack of clinical benefits (82). Pamufetinib, a VEGFR, PDGFR, and HGFR inhibitor, showed acceptable safety in a phase 2 trial (83). Similar to Nintedanib, Pamufetinib also inhibits both PDGFR-α and PDGFR-β (29), however, the targeted inhibition of PDGFR-β but not PDGFR-α attenuates fibrosis (84) in bleomycin-treated mice. Additionally, some tyrosine kinases like VEGFR and FGFR exhibit isoform-specific, context-dependent profibrotic or antifibrotic effects which make growth factor inhibition increasingly complex.

TGF-β remains a key therapeutic target upstream of PI3K/Akt, though its inhibition poses challenges due to adverse effects, including heart valve lesions and skin cancers (85–87). Indirect approaches like integrin inhibition with Bexotegrast, a dual αvβ6/αvβ1 inhibitor, have shown promise, reducing lung function decline in a phase 2 study with no severe adverse events (88). Longer and larger clinical trials are awaited to conclude positive clinical outcomes. Another novel strategy to indirectly inhibit TGF-β signaling involves targeting lysyl oxidase–like 2 (LOXL2), an enzyme restricted to fibroblasts. Inhibition of LOXL2 enzymatic activity induces its auto-oxidation, leading to the creation of a metabolite that directly inhibits TGF-β receptor I (TβRI) kinase (89). Although TGF-β is the main driver of fibrosis, *in vivo* deletion of TGF-β is shown to exacerbate inflammation, hence it is crucial to consider drug design, dosage and strategies for targeting TGF-β. Downstream of PI3K/Akt/mTOR, drugs targeting NFκB, GSK-3β (90) and p706Sk have been developed, though not all have been tested in IPF models (91). Specifically, ACT001, an NFκB inhibitor, was the latest of NFκB inhibitors that demonstrated anti-fibrotic activity in fibroblasts from IPF patients (92).

### 3.4 Senolytics in IPF

Despite the pervasive role of senescence in IPF, current standard-of-care drugs have no effect on senescence markers in IPF tissues (93), highlighting the possibility of a more direct approach. Senolytics, a class of drugs targeting senescent cells, showed promising preclinical and early clinical trial results in IPF patients (20). Quercetin attenuated bleomycin-induced pulmonary fibrosis, by reducing senescence markers like p21 and SASP, and inhibiting apoptosis resistance via modulating Akt activity (94). A first-in-human study with Quercetin and Dasatinib demonstrated selective senescent cell elimination, reduced markers, and modest functional improvement, supporting their potential as IPF therapies (95). Although, larger, randomized clinical trials are necessary to further validate this data, given that mTOR inhibitors have also directly demonstrated reduction in senescence markers, it would be interesting to evaluate the effects of more diverse “senolytic cocktails” targeting both senescence and fibrosis. Senolytics may also complement PI3K inhibitors, offering a synergistic approach to disrupt the profibrotic cycle. Furthermore, other classes of anti-senescence drugs, including SASP inhibitors and drugs targeting senescent pathways such as Navitoclax could be included in these drug cocktails to develop newer therapies. Considering that shortened telomeres are linked to higher risks of IPF, telomerase activators such as TA-65 (96), which counteract telomere shortening linked to IPF progression could be studied in IPF, alone and in combination with inhibitors of PI3K/Akt.

## 4 Roadblocks of PI3K/Akt inhibition in IPF and biomarker-driven solutions

The systemic toxicities of oral PI3K/Akt inhibitors, including gastrointestinal effects, hyperglycemia, and skin rashes, have driven next-generation approaches against the PI3K/Akt pathway such as Akt degraders, inhalation-based drug delivery, prodrugs, and fibroblast-specific conjugates to enhance target selectivity and reduce adverse effects. However, whether such next-generation drugs will not face the same challenges as older ones still needs further clinical experimentation. Despite the promising improvements in safety and efficacy offered by the new class of mTOR inhibitors like Gedatolisib over existing drugs in breast cancer (74), if repurposed to IPF, chronic PI3K inhibition may still lead to unexpected compensatory activation of alternative pro-fibrotic pathways such as Mitogen-Activated Protein Kinase (MAPK) signaling (97). These pathway redundancies could be addressed by Akt degraders like INY-03-041, which was initially developed for oncology, and leverages short-term, high-intensity therapy to achieve rapid systemic Akt depletion, suppressing tumor growth. However, while cancer patients may tolerate these effects during short-term therapy, IPF patients require long-term treatment. These obstacles could be overcome by considering newer routes of administration, intermittent dosing strategies and biomarker detection to monitor early signs of toxicity and resistance to treatment.

In addition to pre-existing issues, these therapeutics will also face new challenges arising from their novelty, such as the inhaled route of administration, prodrug formulations, or unique structures like fibroblast-specific conjugates. Inhaled formulations could enhance local drug bioavailability while minimizing systemic toxicity, as shown by a clinical trial with inhaled pirfenidone (98) but this would bring about inhalation-based challenges such as limited biodistribution due to reduced airflow in areas of severe fibrosis and reduced efficacy due to poor lung penetration. Next, prodrug formulations are a promising approach for minimizing toxicities, but they could generate highly reactive intermediates, increasing the risk of adverse effects, such as DNA damage or mitochondrial dysfunction. Drugs with unique conjugates in their structures like FAPL-PI3Ki1, aim to restrict drug action to fibrotic lung tissue by binding to FAP, whose expression is limited to lungs, thus improving precision and reducing systemic toxicity. However, FAP expression varies across different fibrotic foci and is not uniformly upregulated in all myofibroblasts (99), potentially leading to incomplete pathway inhibition, and subsequently, in residual fibrosis progression despite treatment.

These challenges pose significant hurdles that could delay drug development, but rather than obstacles, they should be seen as critical checkpoints that refine the drug development strategy. Major concerns such as target engagement, response to treatment, resistance mechanisms, and toxicities related to PI3K inhibitors could be better addressed with biomarker-driven patient monitoring. In this regard, response and resistance biomarkers are integral to future clinical trial design. IPF is a highly heterogeneous disease with multiple pathogenic drivers, and the absence of PI3K mutations suggests that its activation in IPF is more likely due to signaling dysregulation rather than genetic alterations. Moreover, disease progression is highly variable and some patients experience acute lung function decline with rapid fibrosis, while others may remain stable for years without significant deterioration. Thus IPF-specific molecular biomarkers would facilitate classification of patient subgroups most likely to benefit from therapies like PI3K inhibitors, Akt degraders or senolytic cocktails. Considering trials with current standard of care, for monitoring Pirfenidone efficacy, serum surfactant protein (SP)-D, a protein released by damaged AECs is a validated pharmacodynamic biomarker with limited prognostic value, while SP-A and KL-6 have been tested as efficacy biomarkers for both Nintedanib and Pirfenidone in a single-center retrospective study (100, 101). However, these biomarkers reflect alveolar epithelial injury but do not capture PI3K/Akt-specific pathway activity or therapeutic response. This makes it even more crucial to establish biomarkers that specifically reflect PI3K/Akt pathway activation and response to inhibition.

Some biomarkers used to assess PI3K inhibitors in cancer could potentially be translated to IPF, given their shared mechanisms. The Omipalisib trial has already demonstrated the feasibility of using p-Akt levels in bronchoalveolar lavage (BAL) fluid macrophages to track target engagement in IPF patients, indicating its relevance as a transferable biomarker (65). Pharmacodynamic biomarkers of PI3K inhibition relevant in tumors—such as phosphorylated proteins like p-PRAS40, p-S6 kinase, and p-4EB, metabolic markers like glucose metabolism, GLUT1, and GLUT4, or imaging biomarkers like ^18^F-FDG-PET (102, 103) —could also have relevance in IPF. Additionally, computational analyses of large IPF datasets could identify new biomarkers, although thus far they have primarily been used to identify diagnostic or predictive markers, rather than prognostic biomarkers tailored for pathway-specific therapies. To date, no single biomarker readout has clear prognostic value over the treatment course. Since a standard-of-care has been established for IPF, new clinical trials cannot ethically incorporate a true placebo. Thus, a composite biomarker framework for monitoring PI3K inhibitor efficacy in IPF, combining endpoint markers like Forced Vital Capacity with transcriptional biomarkers (like FOXO3A), senescence-associated markers (p21, SA-β-gal), and phosphorylation-based readouts would enable dynamic monitoring of patient response, drug efficacy and disease progression. Despite the added complexity of standard-of-care, as more PI3K inhibitors are tested in clinical settings, integrating computational analyses of large-scale patient data in the study design will progressively address the gap in efficacy biomarkers specific to IPF. This strategy will enhance biomarker discovery and trial design efficacy, accelerating the translation of PI3K inhibitors from bench to bedside.

## 5 Conclusion

IPF is a chronic, interstitial lung disease marked by significant patient heterogeneity and a lack of relevant biomarkers to guide drug development. Over the years, considerable progress has been made in understanding the mechanisms driving IPF, particularly the dysregulation of the PI3K/Akt pathway. While recent advances in selective PI3K inhibitors, Akt degraders, and dual PI3K/mTOR inhibitors offer promising therapeutic potential, challenges such as selectivity and systemic toxicities remain significant barriers to their clinical application. Innovative strategies, such as inhalation-based prodrugs [e.g., KITCL27 (75)] and fibroblast-specific conjugates [e.g., FAPL-PI3Ki1 (76)], represent important steps toward reducing systemic toxicity while enhancing therapeutic efficacy.

Senescence is increasingly recognized as a key driver of IPF, with PTEN loss and PI3K/Akt hyperactivation exacerbating senescence in alveolar epithelial cells and fibroblasts, forming a feedback loop that accelerates disease progression. Senolytics, such as Dasatinib and Quercetin, offer a transformative approach by selectively eliminating senescent cells, reducing fibrosis and inflammation in preclinical studies. Addressing IPF requires a multifaceted strategy combining targeted therapies like PI3K inhibitors with interventions tackling aging-related mechanisms, including mitochondrial dysfunction and telomere attrition. Exploring such combination therapies may provide greater benefits by addressing multiple aspects of IPF pathogenesis. While current treatments remain limited, optimizing drug formulations, developing better biomarker-driven patient selection strategies, and combining these therapies with other antifibrotic agents may help overcome these hurdles and improve treatment outcomes in IPF.
